# Bevacizumab for Macular Serous Neuroretinal Detachment in Tilted Disk Syndrome

**DOI:** 10.1155/2010/970580

**Published:** 2010-11-30

**Authors:** Paolo Milani, Alfredo Pece, Luisa Pierro, Patrizio Seidenari, Paolo Radice, Antonio Scialdone

**Affiliations:** ^1^Ospedale Fatebenefratelli-Oftalmico, Corso di Porta Nuova 23, Milan, Italy; ^2^Ospedale Vizzolo Predabissi, Via Pandina 1, Melegnano, Milano, Italy; ^3^Ospedale San Raffaele, Università Vite e Salute, Via Olgettina 60, Milano, Italy

## Abstract

*Background*. Tilted disc syndrome (TDS) is a congenital anomaly characterized by “tilting” of the optic disc tipycally associated with myopic astigmatism, visual field defect, inferior staphyloma, and retinal pigment epithelium atrophy. Associated complications such as macular serous neuroretinal detachment are well described; however, ideal therapy for such complication is unknown. *Methods*. One interventional case report is hereby described. A patient affected by macular serous neuroretinal detachment-complicated tilted disk syndrome underwent a complete ophthalmic examination. Optical coherence tomography and fluorescein angiography were taken at baseline and at scheduled visits. Two intravitreal treatments of bevacizumab (avastin, 1.25 mg/0.05 mL) were performed at monthly interval. *Results*. At scheduled visit, one month after the second injection, OCT depicted persistence of neuroretinal detachment. Best-corrected visual acuity remain stable as well as metamorphopsia and functional discomfort. *Conclusion*. Clinical evidence of this brief interventional case report indicates that one patient affected by recent serous macular detachment-complicated TDS did not benefit from 2 consecutive monthly intravitreal Avastin treatments. Best-corrected visual acuity remained stable over a total observation period of 6 months.

## 1. Introduction

Tilted disc syndrome (TDS) is a congenital anomaly characterized by a nasal-to-temporal “tilting” of the optic disc associated with inferonasal crescent, myopia, myopic astigmatism, visual field defect, inferior staphyloma, and retinal pigment epithelium (RPE) atrophy [1–4]. The syndrome is associated with complications such as macular serous neuroretinal detachment [5, 6] or choroidal neovascularization [7].

## 2. Matherials and Methods

This case report is about a 70-year-old woman who presented with loss of visual acuity (VA) in the left eye, with metamorphopsia dating from a few days before. 

The patient's eyes were carefully examined including best-corrected VA (BCVA) testing (Snellen equivalent), optical coherence tomography (Stratus OCT, Carl Zeiss, Dublin, CA, USA) and fluorescein angiography (Topcon Corp., Japan). 

After obtaining informed consent, off-label bevacizumab (Avastin, 1.25 mg/0.05 mL) was injected intravitreally twice at monthly interval, in the operating theatre through a 30 gauge needle at 3.5 to 4 mm of inferotemporal limbus.

## 3. Results

**Figure 1:**
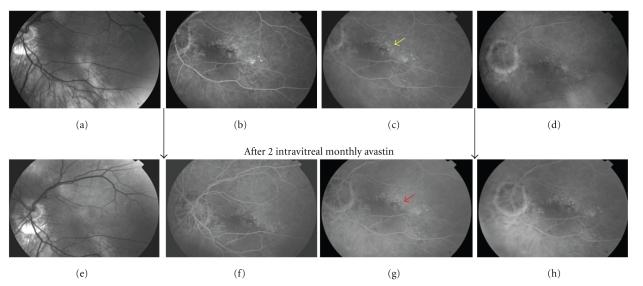
(a), (b), (c), (d): imaging before intravitreal bevacizumab. (e), (f), (g), (h): imaging after intravitreal bevacizumab. Red free picture (a) shows tilting of optic disk with inferior staphyloma. RPE pigmentary changes are evident along the staphyloma area. In early mid-phases of fluorescein angiography (b, c) an hyperfluorescent dot (yellow arrow) becomes evident over a background of diffuse oblique-oriented RPE atrophy that typically overlies the staphyloma. At 5-minutes late phase (d) leaking point intensity dissolves into a diffuse vanishing hyperfluorescence. After treatment the primitive leaking dot (red arrow) is not detectable in early (f), middle (g), or late phases (h) of fluorangiography.

When referred to our attention patient's best corrected VA was 20/100 (refraction error: +0.50–3@180D). Fundus examination showed tilted insertion of the optic disk with atrophic peripapillary changes associated with inferior staphyloma. Fluorescein angiography (FA) disclosed a foveal diffuse RPE atrophy/distrophy and a small hyperfluorescent spot with little leakage in the middle-late phases of the examination (yellow arrow, Figure 1, top stripe), corresponding to the focal RPE leaking point. 

**Figure 2:**
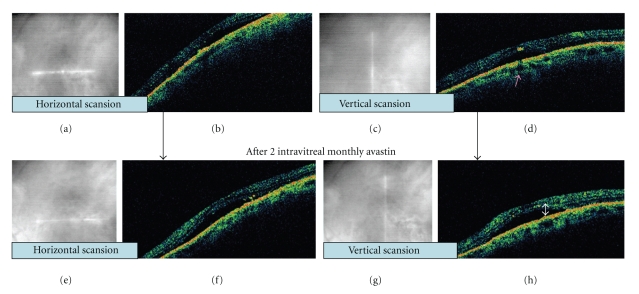
(a), (b), (c), and (d): imaging before intravitreal bevacizumab. (e), (f), (g), and (h): imaging after intravitreal bevacizumab. (a), (c), (e), and (g): infrared imaging shows macular position of OCT scanner at infra-red picture. Serous neuroretinal detachment is well evident in both vertical and horizontal scansion, before (b, d) and after bevacizumab injection (f, h). Presumed focal defect in RPE corresponding to the active leaking point is indicated by the pink arrow. Amount of serous fluid (white arrow) leaking from underling RPE-choriocapillary layer is tomographically unchanged.

OCT evidenced a foveal serous neuroretinal detachment (Figure 2, top stripe) in both vertical and horizontal scansion.

At scheduled visit, one month after second injection, complete imaging was repeated.

OCT depicted persistence of neuroretinal detachment (Figure 2, bottom stripe) whilst FA (Figure 1, bottom stripe) showed disappearance of previous hyperfluorescent spot. Entity, distribution, and dimension of RPE atrophy/dystrophy area unchanged and primitive leaking dot was completely angiographically silent.

Best-corrected VA remained 20/100 and metamorphopsia were stable. The patient refused additional treatments.

Six months after treatment, the macular detachment remained per OCT.

## 4. Discussion

Subretinal leakage in myopic eyes with TDS remains a poorly recognized disease entity and a relatively young disease, as first reports were published in 1998 [5]. It is extimated to occur in 41% of TDS cases [7]. Although OCT technology is a useful aid in diagnosis and followup, the natural history and pathophysiology of serous macular detachment are not well understood. Anti-VEGF therapy with intravitreal injection of ranibizumab and pegaptanib has been approved for neovascular age-related macular degeneration. Additionally, bevacizumab is utilized worldwide as an off-label therapeutic option in many other retinal disease such as pathologic myopia and central serous chorioretinopathy (CSC) [8], due to the low cost of the drug, with encouraging results. 

CSC is a well-characterized disease with typical findings including subretinal leakage similar to aspects of TDS-associated serous macular detachment. Retinal pigment epithelium dysfunction has been suggested as a possible primitive step in both pathological conditions. Intravitreal bevacizumab has been reported in 5 patients as a new option in the treatment of central serous chorioretinopathy [8] and was associated with visual improvement and reduced neurosensory detachment without adverse events. 

In our patient, laser photocoagulation of the leakage was not possible in our opinion because of its foveal position, so we decided to employ 1.25 mg intravitreal avastin as off-label treatment. Following bevacizumab, repeated FA documented the disappearance of the early leaking point (red arrow, Figure 1, bottom stripe), but the serous fluid did not reduce on OCT (Figure 2, bottom stripe). This is difficult to explain, but some discordance is known between FA and OCT imaging [9]. In our patient, it is noteworthy that persistent neuroretinal detachment was more strongly tied to functional recovery than angiographic pinpoint activity.

As the prognosis of untreated TDS-related macular serous detachment is not well defined, we can postulate that it can wax and wane similarly to chronic CSC or perhaps that intravitreal avastin may have some influence on closing the leaking dot.

It may have been illustrative to investigate the effects of further intravitreal treatments, but the patient experienced no BCVA improvement and declined additional therapy; macular serous detachment persisted at 6-month followup.

In conclusion, the ideal treatment for macular serous detachment in TDS remains unknown. In this single case, Avastin as employed did not appear to influence BCVA outcome. 

More data about Avastin and other VEGF inhibiters in the treatment of serous macular detachment-complicated TDS are required to further define prognosis and efficacy.
